# Underwater Attachment of the Water-Lily Leaf Beetle *Galerucella nymphaeae* (Coleoptera, Chrysomelidae)

**DOI:** 10.3390/biomimetics7010026

**Published:** 2022-02-14

**Authors:** Constanze Grohmann, Anna-Lisa Cohrs, Stanislav N. Gorb

**Affiliations:** Department of Functional Morphology and Biomechanics, Zoological Institute, Kiel University, Am Botanischen Garten 9, 24118 Kiel, Germany; cgrohmann@zoologie.uni-kiel.de (C.G.); anna.cohrs92@hotmail.com (A.-L.C.)

**Keywords:** adhesion, underwater, contact angle, insect, biomechanics, locomotion

## Abstract

While the reversible attachment of artificial structures underwater has moved into the focus of many recent publications, the ability of organisms to walk on and attach to surfaces underwater remains almost unstudied. Here, we describe the behaviour of the water-lily leaf beetle *Galerucella nymphaeae* when it adheres to surfaces underwater and compare its attachment properties on hydrophilic and hydrophobic surfaces underwater and in the air. The beetles remained attached to horizontal leaves underwater for a few minutes and then detached. When the leaf was inclined, the beetles started to move upward immediately. There was no difference in the size of the tarsal air bubble visible beneath the beetles’ tarsi underwater, between a hydrophilic (54° contact angle of water) and a hydrophobic (99°) surface. The beetles gained the highest traction forces on a hydrophilic surface in the air, the lowest on a hydrophobic surface in air, and intermediate traction on both surfaces underwater. The forces measured on both surfaces underwater did not differ significantly. We discuss factors responsible for the observed effects and conclude that capillary forces on the tarsal air bubble might play a major role in the adhesion to the studied surfaces.

## 1. Introduction

In adhesion science, the topic of underwater attachment has moved into the focus of many publications in recent years. Permanent underwater adhesion using glues is well known for numerous aquatic animals [1,2] and this knowledge was even transferred to the synthesis of biomimetic glues [3,4,5,6]. Also, reversible artificial adhesive systems based on surface nano- and microstructures for underwater application were recently produced [7,8,9]. However, reversible adhesion by animals underwater was the concern of only a few studies. Hosoda and Gorb (2012) studied beetles inhabiting a terrestrial environment [10]. Other researchers focused on the underwater adhesive performance of the hair-like structures of, e.g., diving beetles or mussels, without considering the attachment of the organism as a whole [11,12]. In summary, many questions on the attachment of organisms remain unanswered. For example, the adhesion to wet substrates as well as the roles of capillary adhesion and nanobubbles still need to be clarified [13].

Smooth and hairy attachment devices occur in different insect taxa, but nubby structures can also be found [14,15]. Adult beetles usually possess a hairy attachment system [16] and the attachment is promoted by the secretion of a mixture of hydrocarbons, fatty acids, and alcohols onto the contact area [17,18]. Different physico-chemical principles rule underwater adhesion compared with attachment in the air. Hosoda and Gorb (2012) discovered that, due to the hairy microstructure of the beetles’ tarsi, an air bubble is stably kept underwater beneath the feet of the green dock beetle *Gastrophysa viridula* [10]. They postulated that these bubbles de-wet substrates and allow direct contact between the pad fluid and the dried solid substrate surface. Additionally, the air bubble itself produces capillary adhesion at its perimeter. However, *G. viridula* is a terrestrial beetle that lives and feeds on the common sorrel *Rumex acetosa* and can only submerge its feet underwater during heavy rain. In the present study, we aimed at studying the reversible underwater attachment system in a species that depends more strongly on a good attachment performance underwater. We have therefore chosen the limnic water-lily leaf beetle *Galerucella nymphaeae* (Linnaeus 1758), which lives and forages on floating leaves of the waterlilies *Nuphar* sp. and *Nymphaea* sp. [19]. This beetle species is regularly exposed to wet and submerged surfaces, i.e., the leaves of its host plants are submerged or flooded, for example, by water birds trampling them down. Especially after rain, patches filled with water often remain on the leaves’ surfaces for quite a long time.

In the present paper, we first asked how *G. nymphaeae* behaves when it is submerged while attaching to a surface. Second, we tested whether the beetles’ tarsal air bubbles differ in size on hydrophilic (54° contact angle) and hydrophobic (99° contact angle) surfaces. Third, we assessed the traction forces of *G. nymphaeae* on these two types of surfaces. Finally, we measured the buoyancy forces of *G. nymphaeae* and *G. viridula* and included these data in our discussion of the factors that might be responsible for the observed underwater adhesion mechanism.

## 2. Materials and Methods

### 2.1. Tarsal Morphology

*Galerucella nymphaeae* beetles were collected from floating leaves of *Nuphar lutea* at a small artificial pond located in the botanical garden of Kiel University, Germany in July and August 2016. *Gastrophysa viridula* beetles were collected from *Rumex obtusifolius* plants in the surrounding areas of Kiel during the same months. For scanning electron microscopy (SEM), tarsi of *G. nymphaeae* were air-dried, attached to the SEM stubs, and sputtered with a ~20 nm thick layer of gold-palladium. Images were taken with a SEM Hitachi S4800 (Hitachi High-Technologies, Tokyo, Japan) at an accelerating voltage of 3–5 kV.

### 2.2. Behaviour Underwater

Adult individuals of *G. nymphaeae* were observed on floating leaves of *N. lutea* in the botanical garden of Kiel University. We documented some general behavioural patterns of beetles in their natural environment.

#### 2.2.1. Horizontal Leaf

To assess the behaviour of *G. nymphaeae* when it is pressed down underwater, floating leaves of *N. lutea* with a beetle attaching to its upper side were pressed 10 cm deep underwater. The leaves were held horizontally the entire time. The behaviour of the beetles was observed and the period of time until they detached from the leaves was measured. We took the data of seven beetles that were already running when the lily pad was pushed down underwater. A further seven individuals were sitting still when pushed underwater. Each beetle was measured three to four times; the recovery time between two runs was one minute. In total, 25 measurements were recorded with running beetles and the same amount with beetles standing still.

#### 2.2.2. Sloped Leaf

A similar kind of experiment was performed with leaves that were pressed down at an angle of 60° to the water’s surface. We observed and recorded the behaviour of nine beetles that were running and a further nine individuals that were standing still. Each beetle was tested once.

For the following experiments in the lab, adult individuals of *G. nymphaeae* were collected in the botanical garden of Kiel University. We kept them underneath a piece of *N. lutea* leaf in a Petri dish containing a moist tissue. The tissue and the leaf were renewed after a couple of days. When no experiments were being performed, the Petri dish was stored in a fridge at approximately 10 °C.

### 2.3. Experiment 1: The Formation of the Subtarsal Air Bubbles on Different Surfaces

To determine the size of the air bubble trapped beneath the tarsi of *G. nymphaeae* underwater, we allowed the beetles to attach to a glass slide that was then pushed into a Petri dish filled with tap water. We immediately took images from underneath the beetles’ feet with a stereo microscope (Leica MZ 205A, Leica GmbH, Wetzlar, Germany) that was mounted upside down. The Petri dish was fixed above the objective lens of the inverted microscope. Using coaxial illumination, the contact area between the tarsi or the air bubble and the glass slide could be seen and photographed through an ocular camera (BMS Eyepiece & C-mount camera, 5 Megapixel, Breukhoven, The Netherlands) (Figure 3). We compared the area of the air bubbles in beetles standing on hydrophilic surfaces with those on hydrophobic surfaces. To make glass slides hydrophilic or hydrophobic, we silanised them with (3-Aminopropyl)triethoxysilane (Carl Roth, Karlsruhe, Germany) and Dichlorodimethylsilane (Merck Schuchardt OHG, Hohenbrunn, Germany) and gained contact angles of water on these surfaces of 54.22° ± 2.30° and 99.38° ± 1.87°, respectively (*n* = 20 each; measured with a contact angle measurement device OCA 200, Dataphysics, Filderstadt, Germany). To measure the area of the bubble, we used Photoshop CS 5 (Adobe Systems GmbH, Munich, Germany).

In some cases, the bubbles trapped under the beetles’ feet were connected to a bubble that surrounded the entire body. This occurred in equal numbers (*n* = 8) on both surfaces and often when the beetle’s tarsus was close to its body. We excluded these cases from our dataset.

We took images of up to three different, randomly chosen legs of 21 *G. nymphaeae* individuals on both surfaces, summing up to a sample size of 34 for the 54° surface and 28 for the 99° surface. We then measured the time until the beetles detached from the different glass surfaces. Finally, we calculated how the size of the air bubble changed from the beginning of the experiment compared with the moment directly before beetle detachment. This last experiment was performed for 19 and 17 specimens on the CA = 54° and CA = 99° surfaces, respectively.

### 2.4. Experiment 2: Traction Force Measurements of the Beetles Walking on Different Surfaces

To test the beetles’ attachment abilities on different surfaces underwater and on land, we used a load cell force transducer (BIOPAC systems, Goleta, CA, USA) with a 25 g sensor. The sensor was clamped vertically above the test surface. One end of a human hair was fixed to the sensors’ end and the other end was glued to the elytra of *G. nymphaeae* with the aid of a droplet of molten beeswax. When a beetle walked, it pulled the sensor via the hair. The resulting force was recorded during a 60 s long pulling period and the peak force was determined and used for later analyses.

We tested pulling forces that the beetles generated on hydrophilic and hydrophobic glass slides in a random order. Contact angles of water on these surfaces averaged 54.22° ± 2.30° (mean ± sd, *n* = 21) and 99.38° ± 1.87°, respectively (measured with the contact angle measurement device OCA 200, Dataphysics, Filderstadt, Germany). All but two beetles (*n* = 33) were tested twice on each surface, in air (*n* = 33) and with the surface and the beetles submerged in the tap water (*n* = 31).

### 2.5. Experiment 3: Measurement of the Buoyancy Force

In our discussion, we compare the traction forces of *G. nymphaeae* with previous findings for *G. viridula* [10]. In order to assess whether differences in the traction forces between these two species are caused by their different buoyancy forces, we measured the forces that are needed to push individual beetles of *G. nymphaeae* and *G. viridula* underneath the water’s surface. We therefore used a wire loop and measured the forces with a load cell force transducer with a 10 g sensor (BIOPAC systems, Goleta, CA, USA). We used distilled water that we degassed beforehand in an ultrasonic bath (Bandelin Sonorex RK 52, BANDELIN electronic, Berlin, Germany) at a frequency of 35 kHz. Due to the agility of the beetles, we froze them at −80 °C for at least 10 min and thawed them at room temperature immediately before the measurements. The specimens were carefully laid on the water surface and pushed down in a controlled manner using a motorised micromanipulator that moved the sensor and the wire loop at the speed of 600 µm per second. We measured the maximal forces that occurred (i) while the beetles were pushed down and deformed the water surface but still had contact to the air and (ii) when the beetles’ bodies were completely underwater. The same individuals were pushed down in a 0.1% solution of the surfactant Triton X (Sigma-Aldrich Chemie GmbH, Steinheim, Germany) as a control. The Triton X solution was also degassed in the ultrasonic bath with the same frequency prior to the experiment. We measured forces of 20 individual beetles of each species in distilled water and directly afterwards in the aqueous solution of Triton X.

## 3. Results

### 3.1. Tarsal Morphology

**Figure 1:**
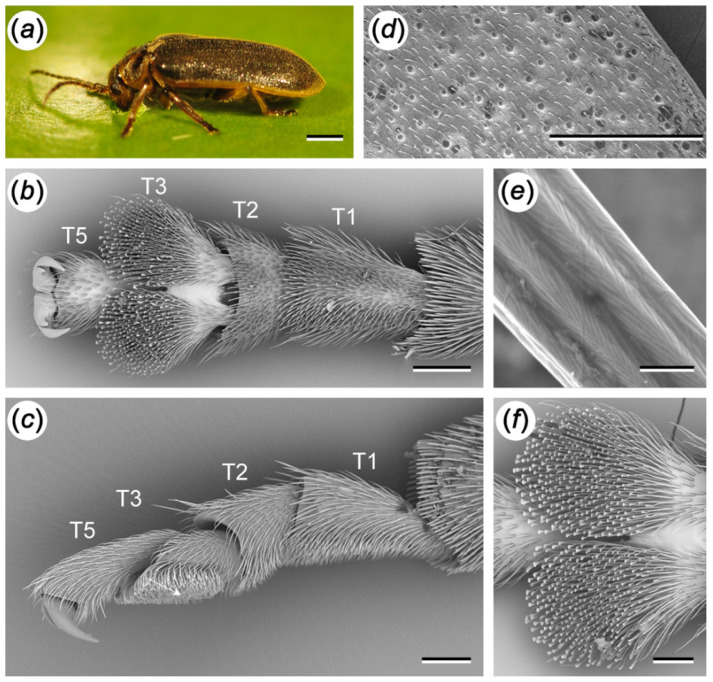
Appearance and tarsal attachment organs of adult *G. nymphaeae*. (**a**) *G. nymphaeae* on the water-lily surface. (**b**) Tarsus, ventral. (**c**) Tarsus, lateral. (**d**) Elytra. (**e**) Hair coverage on the elytra. (**f**) Third tarsal segment, ventral. (**d**–**f**) SEM images. T1–T5: Tarsal segments; T4 is reduced. Scale bars: (**a**) = 1 mm; (**b**,**c**) = 100 μm; (**d**) = 500 μm; (**e**) = 1 µm, (**f**) = 50 μm. (**a**) Courtesy of Andreas Blankenstein. (**b**,**c**,**f**) Reprinted with permission from ref. [15]. Copyright The Authors of the ref. [15] under the license of Company of Biologists Publication Agreement.

The ventral sides of the first and second tarsomeres of *G. nymphaeae* are covered with long pointed setae, while the third tarsomere bears spatula-shaped setae (Figure 1). We did not detect any differences between the attachment structures of female and male beetles and therefore do not differentiate between sexes in this study (Figure S5, Supplementary Materials). The elytra of the water-lily leaf beetle are covered with long hairs (Figure 1d,e).

### 3.2. Behaviour Underwater

We observed that *G. nymphaeae* avoids walking through wet patches on the leaves. When a beetle falls off into the water, it swims actively to the nearest leaf or to the rim of the pond. When water was sprayed over the beetles to imitate rain, they stopped moving and withstood the “shower”. This applies to formerly running and still-standing beetles.

#### 3.2.1. Horizontal Leaf

When pressing *G. nymphaeae* underneath the water’s surface, a visible bubble encased the elytra of nearly each inactive beetle; active ones had this bubble in roughly 60% of the observations. Underwater, all individuals of *G. nymphaeae* opened their elytra after a while, and a bubble under the abdomen became visible. Independently of the time spent underwater, some of the beetles waggled their legs before detaching from the leaf.

**Figure 2:**
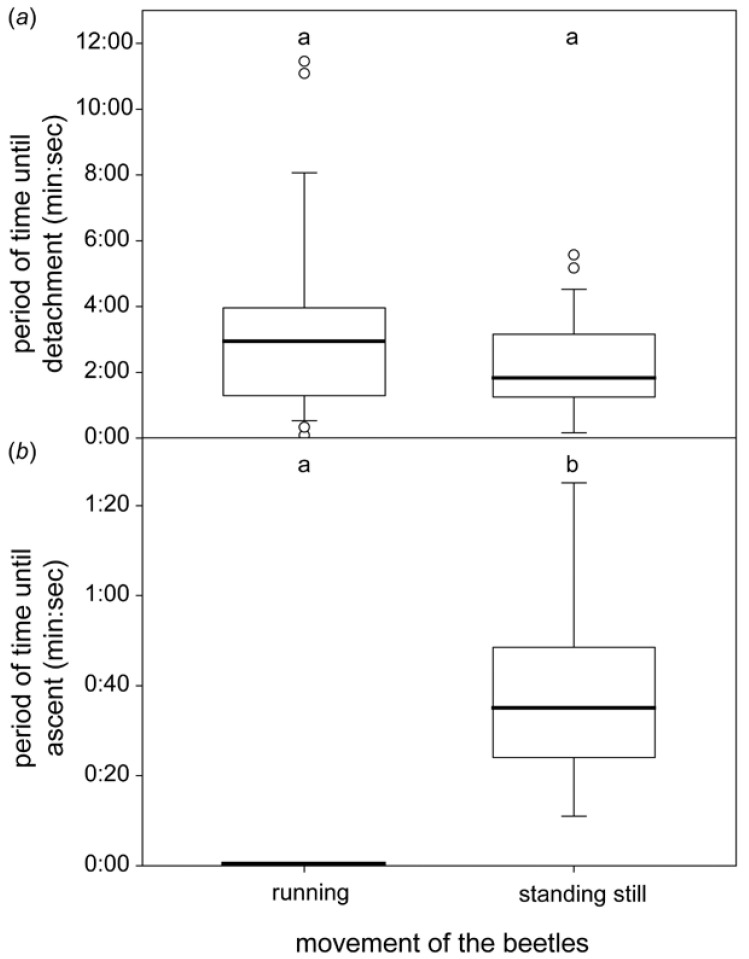
Period of time until *G. nymphaeae* detaches from the horizontal leaf (upper plot) or ascends to the top of the inclined leaf (lower plot) underwater. The plots show the medians (lines within the boxes), 25th and 75th percentiles (ends of boxes), 10th and 90th percentiles (error bars), and outlying values (circles). Different letters above two boxes indicate significant differences between two groups. Upper plot: seven running and a further seven beetles standing still were tested 3–4 times (*n* = 25 for each test). Lower plot: nine running and a further nine beetles standing still were tested once (*n* = 9 for each test). Different letters (**a**,**b**) within each subplot indicate presence of statistically significant difference between samples.

Those beetles that were already running on the leaf in air continued to run when it was pressed into the water, and they detached after an average of 2:57 (1:18–3:58) min. (median value, interquartile range; Figure 2). Only a single beetle detached after more than 5 min underwater, and it did so in three out of four repetitions.

Beetles that stood still in the air remained roughly half as long underwater until they detached (1:49, 1:15–3:10 min), but we recognized a great variation in the time periods even within a single individual. The differences between running beetles and those standing still were not significant (*p* = 0.28, Mann–Whitney Rank Sum Test).

#### 3.2.2. Sloped Leaf

All formerly running beetles started to move upward within 5 s on a sloped water-lily pad underwater (Figure 2). Those that were standing still before started to ascend after 0:35 (0:24–0:49) min.

### 3.3. The Formation of the Subtarsal Air Bubbles on Different Surfaces

**Figure 3:**
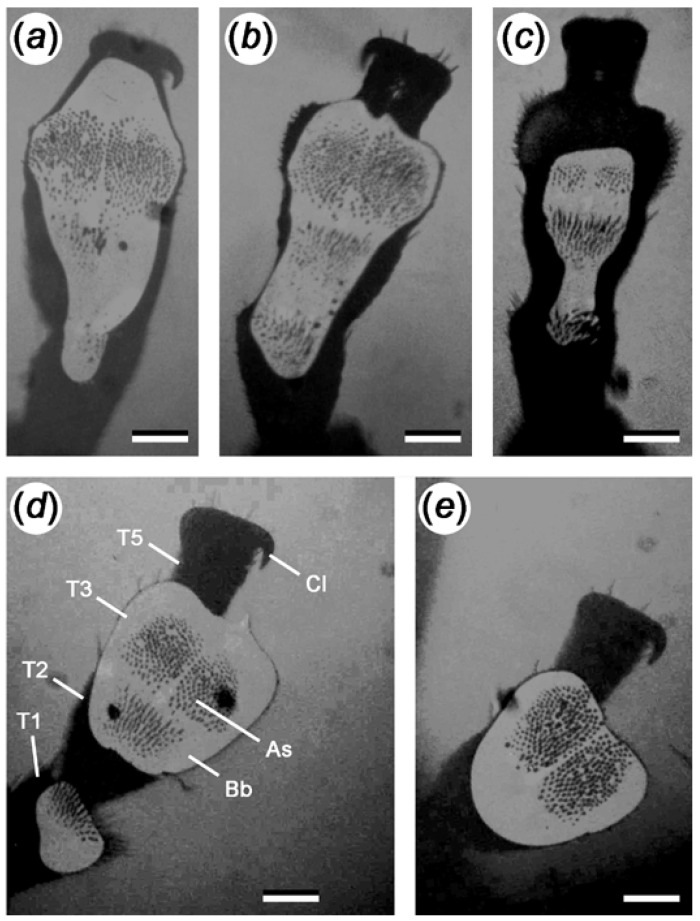
Images of the tarsal air bubble of *G. nymphaeae* beetles standing on glass slides underwater. Due to coaxial illumination, the air appears white and the contact area of attachment hairs on the glass slide appears black. As: Attachment setae. Bb: Subtarsal air bubble. Cl: Claw. T1–T5: Tarsal segments. Scale bars: (**a**–**e**): 0.1 mm.

**Figure 4:**
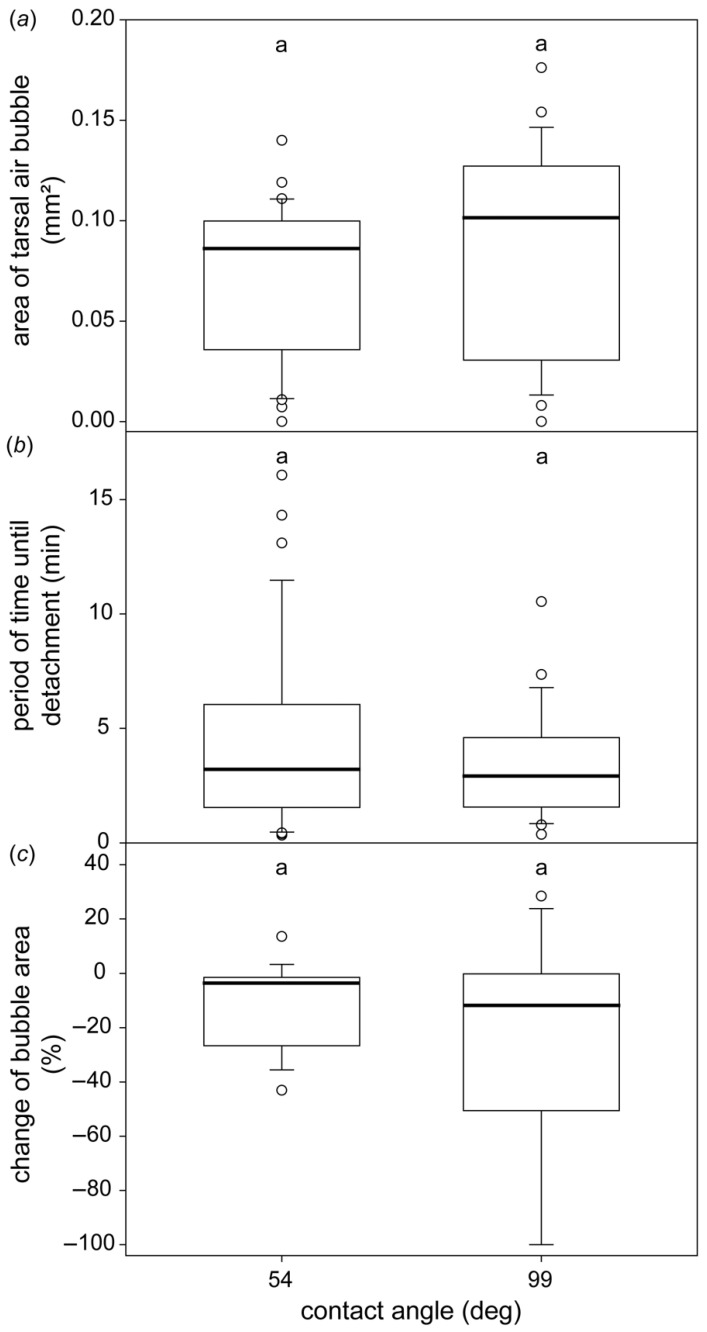
The subtarsal air bubble on different surfaces. Area of the tarsal air bubble taken immediately after *G. nymphaeae* was submerged on hydrophilic and hydrophobic glass slides (**a**), time until beetle detachment (**b**), and difference of the bubble area between the beginning of the experiment and just before the beetle detached (**c**). The plots show the medians (lines within the boxes), 25th and 75th percentiles (ends of boxes), 10th and 90th percentiles (error bars) and outlying values (circles). The same letter above the two boxes in each experiment indicates no significant differences between the two groups. Sample size: 1–3 different randomly chosen legs of 21 beetles; upper and middle plot: *n* = 34 and *n* = 28, lower plot: *n* = 19 and *n* = 17 for the 54° and 99° surfaces, respectively. Same letters (a, a) within each subplot indicate absence of statistically significant difference between samples.

In general, we discovered a great variety of different shapes and sizes of tarsal air bubbles, both between individual beetles and within the same individual (Figure 3). The size of the bubble did not differ significantly on either of the two glass plates with 54° and 99° contact angles, respectively (Mann–Whitney Rank Sum Test, *p* = 0.101; Figure 4). Similarly, the lapse of time the beetles spent underwater until they boosted themselves to the water surfaces and the difference in the bubble size at the beginning and immediately before the beetles detach did not depend on the surface (Mann–Whitney Rank Sum Test, *p* = 0.969 and *p* = 0.547, respectively; Figure 4).

### 3.4. Traction Force Measurements of the Beetles Walking on Different Surfaces

**Figure 5:**
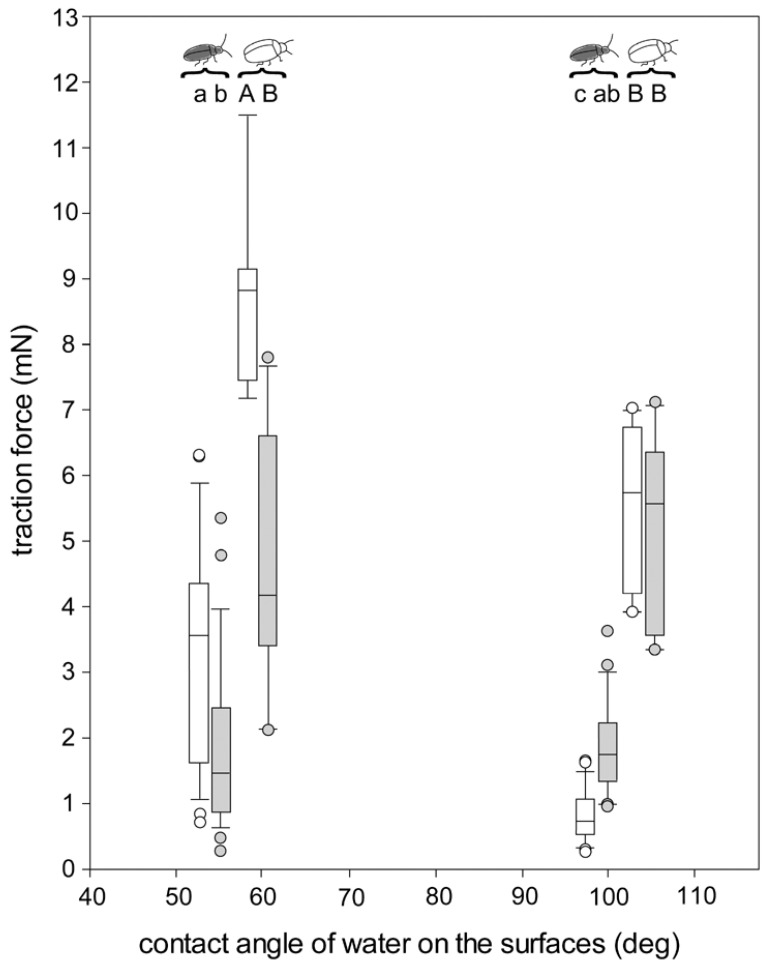
Traction forces of *G. nymphaeae* on different surfaces in air and underwater. For comparison, data for *G. viridula* are added from [10]. The plots show the medians (lines within the boxes), 25th and 75th percentiles (ends of boxes), 10th and 90th percentiles (error bars), and outlying values. White boxes and circles: in air; grey boxes and circles: underwater. Different letters above two boxes (separately within small letters and within large letters) indicate significant differences between two groups (multiple pairwise comparisons). Pairs of boxes at 54° and 99° (black beetle icon): *Galerucella nymphaeae*. Pairs of boxes at 59° and 104° (white beetle icon): *Gastrophysa viridula*. Each beetle (*n* = 33 for *G. nymphaeae*, *n* = 29 for *G. viridula*) was tested once in air and once underwater; two of these *G. nymphaeae* beetles were solely tested in air. Different letters (a, b or A, B) indicate statistically significant difference between samples.

In air, the maximal traction force the beetles gained was almost four times higher on glass slides with a 54° contact angle (3.69 ± 1.92 mN, mean ± sd) compared to slides with a 99° contact angle (1.00 ± 0.81 mN) (Figure 5; One Way Repeated Measures ANOVA with Holm–Sidak pairwise comparisons, *p* < 0.001). In contrast, no significant differences in traction forces were found between the two surfaces when the beetles were underwater (54° surface: 1.79 ± 1.27 mN vs. 99° surface: 1.80 ± 0.78 mN; *p* = 0.975).

### 3.5. Measurement of the Buoyancy Force

When the beetle was pushed down slightly, the tension film of the water surface was deformed and the beetle and/or the air bubble that encased it still had contact with the air. In this situation, the force to push *G. nymphaeae* down was 1.6 times greater than the force needed for *G. viridula* (0.78 ± 0.11 mN and 0.49 ± 0.10 mN, respectively; *p* < 0.001, *t*-test). When there was no longer contact with the air and the beetle was surrounded by water, the force needed to push the beetle down was less than a tenth of the previous value for both species (0.07 ± 0.01 and 0.03 ± 0.01 mN, respectively; *p* ≤ 0.001, Mann–Whitney Rank Sum Test).

When being pushed in the aqueous solution of Triton X, the beetles, especially *G. viridula*, sank almost immediately to the ground of the plastic jar. Within each species, the force needed to press the beetle down did not depend on the beetle’s weight (*G. nymphaeae*: bubble still having contact to the air: R^2^ = 0.05, linear regression, beetle fully encased by water: R^2^ < 0.01; *G. viridula*: R^2^ = 0.02 and R^2^ = 0.06, respectively).

## 4. Discussion

### 4.1. Tarsal Morphology

We found similarly shaped attachment hairs on the tarsi of both sexes of *G. nymphaeae*. While the males of many chrysomelid species have discoidal tips of attachment setae, to securely attach to the females’ smooth elytra during copulation [20], we assume the lack of such specialised hairs is due to the presence of closely spaced hairs on the female and male elytra of *G. nymphaeae* beetles [15].

### 4.2. Behaviour Underwater

We observed no difference whether the beetles were running or standing still; from both initial situations, they continued to run or stand on the leaf for some minutes after it was submerged horizontally into water. This proves that, although the beetles in general avoid flooded patches on leaves, they can attach well and even run underwater. The individuals that were moving tended to walk to the rim of the leaf. This behaviour enables the beetles to grip and climb upward at the leaf’s edge by using the action of their contralateral legs. If a leaf was pushed at an incline underwater, beetles that were running beforehand immediately started to walk and ascend the leaf. Those that were standing started to walk and ascend the leaf in most cases within 1 min. This behaviour is a strategy for escaping from the submerged situation. It remains, however, unclear whether this behaviour is common for chrysomelid beetles or whether it has evolved in *G. nymphaeae*, owing to its limnic habit.

### 4.3. Subtarsal Air Bubbles on Different Surfaces

In contrast to our initial hypothesis, the area of the tarsal air bubble did not differ between hydrophilic and hydrophobic surfaces. Possible differences might be masked by the fact that on each surface, the size of the bubble varied greatly (Figure 3 and Figure 4), and using our technique, we were able to detect and analyse the bubble only under a single tarsus at a time. According to our hypothesis, a larger tarsal air bubble on a hydrophobic surface should prolong the period of time until the beetle detaches from the surface. Similarly, the bubble area should decrease to a larger extent on the hydrophobic surfaces. However, in both cases, we did not detect any differences between the two surfaces (Figure 4). There could be two reasons for this. On the one hand, as already mentioned, possible differences might be covered by the large variability of the bubble shape and volume on a single tarsus. In turn, the bubble size of a single tarsus might be balanced by the bubble sizes below the other five tarsi of the beetle. On the other hand, the period of time until the beetle detaches might be ruled by other factors, such as the amount of oxygen stored in the air bubble encasing the beetle elytra.

### 4.4. Traction Forces on Different Surfaces and Buoyancy

#### 4.4.1. Traction Forces on Hydrophilic and Hydrophobic Surfaces in Air

In air, *G. nymphaeae* revealed stronger traction forces on the hydrophilic compared with the hydrophobic surfaces (Figure 5). Such differences might be attributed to the chemical composition of the attachment fluid that seems to form a stronger contact between an attachment hair and the surface on hydrophilic compared with hydrophobic surfaces [15]. It was previously shown that a film of water is present on hydrophilic surfaces in air. Capillary forces between this water layer and a probe tip lead to enhanced friction forces, depending on the film’s thickness, if compared with a solid–solid contact [21,22]. Additionally, to the fluid’s chemical and physical properties, interactions between the film of water on the surface and the attachment devices could lead to the observed stronger traction forces on the hydrophilic surface compared with the hydrophobic surface in air. We do not expect such a film of water on the hydrophobic surfaces in air, as only at a humidity of >90% is a monolayer of water formed on such surfaces [23].

#### 4.4.2. Traction Forces on the Hydrophilic Surface in Air and Underwater

In contrast, on the hydrophilic surface, *G. nymphaeae* revealed weaker traction forces underwater when compared with those measured in air. As long as hairs attach to a dry de-wetted surface underwater, the role of the fluid should be exactly the same as in air. The fluid resembles the composition of the beetles’ cuticular lipids [17,18] and it is unlikely that the fluid can be adapted to the substrate by the beetle in such a short period of time. Hence, the relatively low traction forces underwater compared with those in air can either be explained by (i) a reduced number of attachment hairs that form contact to the surface, due to the fact that only hairs within the tarsal air bubble can form a contact, (ii) buoyancy forces, reducing beetle load force to the substrate due to air reservoirs in and around the beetles’ bodies, especially on the hairy elytra of *G. nymphaeae*, or (iii) capillary forces at the tarsal air bubble that were found to be negative on hydrophilic surfaces underwater, increasing with an increasing hydrophobicity of the surface [24].

#### 4.4.3. Traction Forces on the Hydrophobic Surface in Air and Underwater

On the hydrophobic surface, however, a contrary pattern occurs for *G. nymphaeae* with stronger traction forces underwater when compared with those in air. The underlying factors for this effect might be as follows. (i) Capillary forces between the tarsi and the water film on the surface should not play a role here, as we expect only a monolayer of water on the hydrophobic surface in air [23]. (ii) A contact reduction between setae and the surface should lead to a decrease in the traction force. (iii) The same result is to be expected due to the presence of buoyancy forces. (iv) The remaining factor that might be responsible for the higher traction forces underwater on the hydrophobic surface, in comparison to the experiment in air, is increased positive capillary forces at the tarsal air bubble.

We performed our experiments with functionalised glass surfaces with water contact angles of 54° and 99°. We can assume that on surfaces with other contact angles, different combinations of factors might play a role in beetle adhesion. For example, on surfaces with extremely low contact angles, the presence of a thick film of water might impede the contact formation between the tarsi and the surface [25]. It was previously shown that traction forces of the chrysomelid beetle *G. viridula* were strongly reduced underwater on a surface with a contact angle of 43° compared with 59°, and structured polymers did not adhere at all underwater to surfaces with a roughly 20° contact angle [10]. On surfaces without such extremely low contact angles, the surface might be totally de-wetted below the air bubbles [10], enabling contact formation.

There are at least two further studies in which the attachment to surfaces with contact angles similar to ours were assessed. For example, our findings for *G. nymphaeae* are almost similar to those previously obtained for *G. viridula* (Figure 5; [10]). However, while we measured higher traction forces underwater, compared with those in air on the hydrophobic surface, no differences were detected for *G. viridula* on this surface. The traction forces of *G. viridula* were in general higher than those of *G. nymphaeae*, probably due to the higher weight of *G. viridula* in air and its lower buoyancy forces underwater. Shear forces of the tokay gecko (*Gecko gecko*) showed a surprising similarity to our results on a 50° glass surface and a 97° polytetrafluoroethylene surface, although, on a 94° surface (octadecyltrichlorosilane self-assembled monolayer formed on the surface of glass), totally different shear forces of geckos were measured [26].

## 5. Conclusions

From our data, we conclude that for the analysed contact angles of 54° and 99°, capillary forces at the tarsal air bubble seem to play a role in the unexpected higher traction forces of *G. nymphaeae* on the 99° surface underwater, when compared to those in air. We must keep in mind that we did not consider many further factors that might play an additional role. These are, for example, the normal force [27], the presence of surface asperities [26,28], the presence and thickness of a water film on the surface [21], and the degrees of hydrophilicity and hydrophobicity. Strong attachment and friction performance underwater, similar to those found in animal adhesive pads, is of importance for biologically inspired solutions in biomedical engineering and wearable flexible electronics, etc. [29,30,31,32,33,34,35].

## Data Availability

Datasets generated during the study are available in the Supplementary Materials.

## Supplementary Materials

The following are available online at https://www.mdpi.com/article/10.3390/biomimetics7010026/s1, Table S1: Period of time until *G. nymphaeae* detaches from the horizontal leaf underwater or ascends to the top of the inclined leaf underwater, Table S2: The subtarsal air bubble on different surfaces, Table S3: Traction force of *G. nymphaeae* in air and underwater, Table S4: Buoyancy forces of *G. nymphaeae* and *G. viridula*, Figure S1: Female (mid leg) and male (hind leg) setae at high magnification, SEM images.
