# Supplementary material for: Underwater Attachment of the Water-Lily Leaf Beetle Galerucella nymphaeae (Coleoptera, Chrysomelidae)
Source: Biomimetics (Basel). 2022 Feb 14;7(1):26. doi: 10.3390/biomimetics7010026 (PMC8883964; doi:10.3390/biomimetics7010026)
Supplement: Supplementary file 1 [file biomimetics-07-00026-s001.zip › biomimetics-1568833-supplementary.pdf]

Under water attachment of the water-lily leaf beetle *Galerucella nymphaeae* (Coleoptera, Chrysomelidae)

Constanze Grohmann, Anna-Lisa Cohrs and Stanislav N. Gorb

Supplementary materials

Table S1: Period of time until *G. nymphaeae* detaches from the horizontal leaf under water or ascends to the top of the inclined leaf under water.

Period of time until detachment (min:sec)		Period of time until ascend (min:sec)	
**Running beetles**	**Beetles standing still**	**Running beetles**	**Beetles standing still**
03:34	01:47	00:00	00:33
11:05	01:43	00:00	00:11
06:03	04:06	00:00	00:20
11:27	01:20	00:00	00:43
00:40	02:34	00:00	01:25
03:59	03:21	00:00	00:35
00:20	01:49	00:00	00:54
03:29	00:10	00:00	00:28
03:20	03:08	00:00	00:38
04:21	03:31		
03:00	00:10		
03:56	01:01		
00:40	00:40		
00:05	00:10		
01:49	03:11		
02:04	05:34		
02:57	05:10		
02:40	01:40		
01:15	02:56		
03:41	02:43		
04:14	02:50		
01:00	01:30		
01:20	01:50		
01:30	01:10		
01:25	01:45		

Table S2: The subtarsal air bubble on different surfaces. Area of the tarsal air bubble taken immediately, when *G. nymphaeae* was submerged on hydrophilic (contact angle of water: 54°) and hydrophobic (contact angle of water: 99°) glass slides, time until beetle detachment and difference of the bubble area between the beginning of the experiment and just before the beetle detached.

Area of tarsal air bubble (mm^2)		Period of time until detachment (min:sec)		Change of air bubble area (%)	
54°	99°	54°	99°	54°	99°
0.107	0.134	03:16	03:59	-43	-45
0.100	0.077	09:48	07:21	-10	-1
0.091	0.103	03:10	02:07	-28	0
0.088	0.102	04:21	00:47	-2	9
0.038	0.119	00:53	01:40	0	28
0.140	0.014	01:54	03:14	-36	-22
0.090	0.146	04:47	01:32	3	-10
0.104	0.104	05:40	01:00	-1	0
0.031	0.089	04:00	01:15	-2	23
0.084	0.176	00:24	03:29	13	-21
0.080	0.138	01:54	06:33	-3	-4
0.100	0.154	07:51	05:47	-3	-12
0.102	0.109	16:04	02:52	-3	-100
0.057	0.101	02:29	04:39	-7	-62
0.119	0.015	01:48	00:51	-9	-56
0.091	0.014	00:31	04:14	-6	-25
0.096	0.081	07:02	04:54	-1	-100
0.111	0.117	01:36	02:56	-27	
0.011	0.076	01:18	04:26	-34	
0.085	0.008	03:10	01:23		
0.097	0.018	06:01	06:43		
0.012	0.016	01:23	01:54		
0.074	0.000	04:26	00:22		
0.028	0.125	13:06	04:10		
0.019	0.092	05:57	10:32		
0.000	0.129	00:20	01:52		
0.007	0.067	01:49	02:05		
0.075	0.128	14:19	02:34		
0.089		06:07			
0.064		09:51			
0.065		02:42			
0.111		00:47			
0.026		00:26			
0.088		03:15			

Table S3: Traction force of *G. nymphaeae* in air and under water. Beetles were tested in both situation on surfaces with contact angle of water of 54° and 99°.

Beetle Nr.	Traction force (mN) in air		Traction force (mN) under water	
	54°	99°	54°	99°
1	3.531	0.533	2.944	1.579
2	1.900	0.564	4.768	1.513
3	3.749	0.521	1.684	1.766
4	1.085	0.285	5.335	1.737
5	2.906	1.091	0.871	1.242
6	0.736	0.299	0.762	1.311
7	3.139	0.880	0.282	1.086
8	2.223	0.736	0.807	1.041
9	4.256	0.741	0.924	2.023
10	1.725	0.590	1.333	3.619
11	7.018	1.039	0.627	0.394
12	4.285	1.475	3.866	2.214
13	4.669	0.523	2.252	1.656
14	5.826	1.629	1.371	0.981
15	3.787	0.740	0.938	1.877
16	5.774	0.966	3.577	2.989
17	0.853	1.283	2.336	2.484
18	3.807	0.593	2.912	1.499
19	1.563	0.342	1.178	1.512
20	3.976	4.578	0.653	1.269
21	6.266	0.865	1.909	0.947
22	3.583	1.021	0.709	1.225
23	4.374	1.119	0.979	1.455
24	1.107	0.646	0.492	2.060
25	1.592	0.548	1.563	2.379
26	2.039	0.369	2.502	1.861
27	5.261	1.153	2.080	2.231
28	3.660	1.048	0.668	2.208
29	1.435	1.021	0.992	3.097
30	6.296	1.638	2.088	2.666
31	6.927	0.708		
32	6.654	2.835		
33	5.645	0.767		

Table S4: Buoyancy forces of *G. nymphaeae* and *G. viridula*.

Forces when the water surface tension film was deformed but the beetles still had contact to the air (mN)		Buoyancy forces when the beetles were surrounded by water (mN)	
G. nymphaeae	***G. viridula***	***G. nymphaeae***	***G. viridula***
0.8418	0.5956	0.0705	0.0274
0.7189	0.5590	0.0808	0.0437
1.0546	0.4539	0.0938	0.0229
0.8991	0.3916	0.1053	0.0566
0.8721	0.4955	0.0938	0.0342
0.8511	0.6525	0.0896	0.0436
0.7166	0.5285	0.0889	0.0362
0.6695	0.4406	0.0775	0.0203
0.7652	0.5982	0.0700	0.0313
0.6411	0.4962	0.0865	0.0309
0.8743	0.4905	0.0687	0.0304
0.5914	0.6341	0.0484	0.0250
0.8266	0.5572	0.0576	0.0158
0.8117	0.4835	0.0440	0.0417
0.6703	0.3771	0.0637	0.0119
0.6338	0.3699	0.0609	0.0367
0.7315	0.4583	0.0495	0.0179
0.8068	0.3794	0.0728	0.0277
0.9204	0.2955	0.0654	
0.7952			

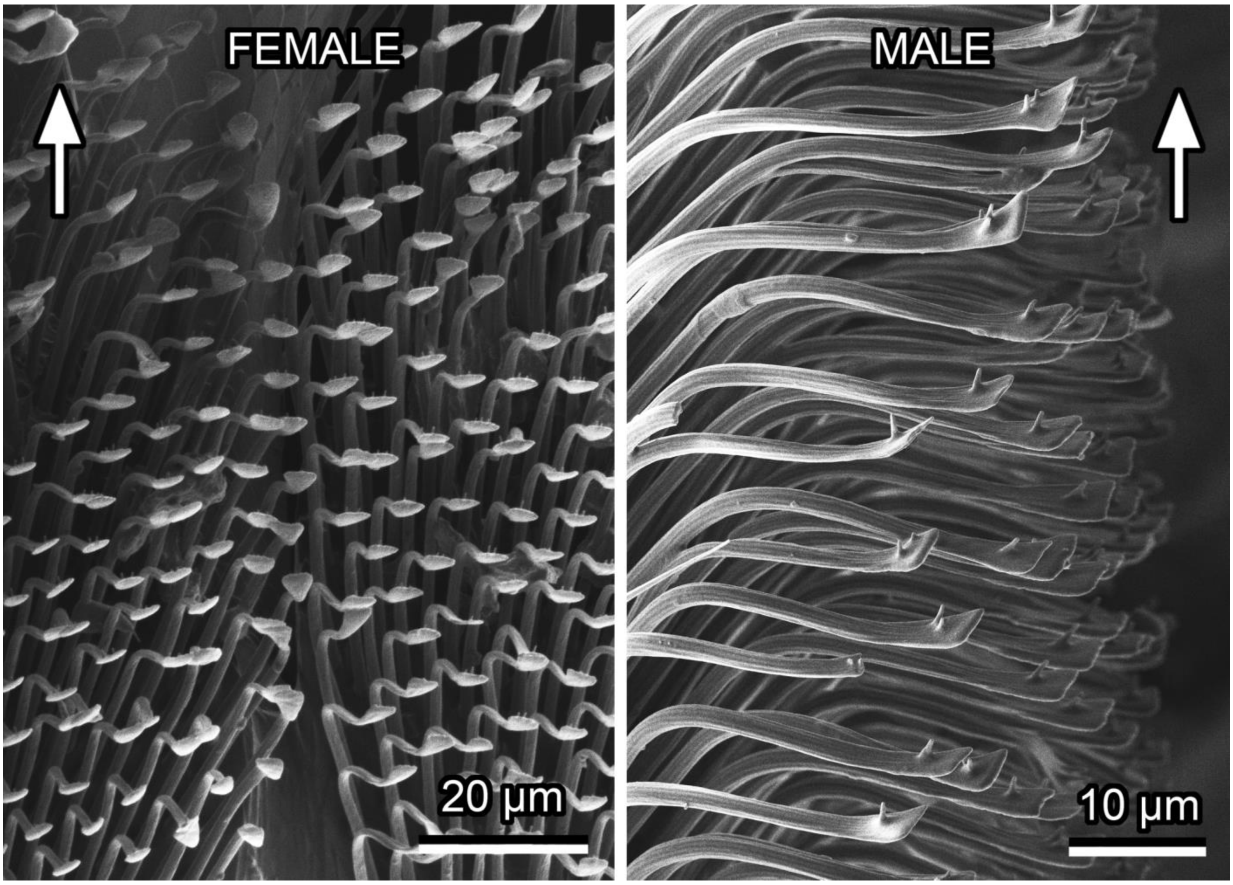

Figure S1: Female (mid leg) and male (hind leg) setae at high magnification, SEM images.
